# Teratoma-free cartilage regeneration using *p21*^−/−^ iPSCs engineered with iCasp9

**DOI:** 10.1093/stcltm/szaf056

**Published:** 2025-11-19

**Authors:** Leila Larijani, Derrick Rancourt, Roman J Krawetz

**Affiliations:** McCaig Institute for Bone & Joint Health, University of Calgary, Calgary, Alberta T2N4N1Canada; McCaig Institute for Bone & Joint Health, University of Calgary, Calgary, Alberta T2N4N1Canada; Department of Oncology, Cumming School of Medicine, University of Calgary, Calgary, Alberta T2N4N1, Canada; McCaig Institute for Bone & Joint Health, University of Calgary, Calgary, Alberta T2N4N1Canada; Department of Cell Biology and Anatomy, Cumming School of Medicine, University of Calgary, Calgary, Alberta T2N4N1, Canada

**Keywords:** cartilage regeneration, iCasp9, induced pluripotent stem cells, tumor formation

## Abstract

Objective: Articular cartilage has limited regenerative capacity due to its lack of innervation, vascularization, and lymphatic vessels. As cartilage is devoid of nerves, injuries often go unnoticed until degeneration leads to pain, reduced function, and ultimately osteoarthritis (OA). Treatment options for cartilage injury, both surgical and nonsurgical, depend on factors like defect size, shape, depth, location, and patient age. Stem cells, particularly their ability to differentiate into chondrocytes, hold promise for cartilage repair, but no therapies have yet gained clinical approval. Recently, induced pluripotent stem cells (iPSCs) have emerged as a potential solution for cartilage regeneration. However, post-transplantation tumorigenesis remains a significant concern. To mitigate this risk, robust quality and safety protocols are needed, alongside safety mechanisms to control iPSC behavior after transplantation.

Design: The iCaspase9 (iCasp9) cell suicide system offers a promising solution, enabling selective elimination of genetically modified cells via apoptosis. We previously demonstrated that the efficiency of iCasp9-mediated killing increases in a p21 mutant background. Since p21 mutations also enhance cartilage repair, we investigated iCasp9-engineered *p21*^−/−^ and wildtype (*p21*^+/+^) iPSCs in a mouse cartilage injury model.

Results: Without iCasp9 activation, both *p21*^−/−^ and *p21*^+/+^ iPSCs formed tumors post-transplantation. In contrast, mice treated with the iCasp9 activator AP20187 showed no tumors. Both *p21*^−/−^ and *p21*^+/+^ iPSCs demonstrated similar cartilage regeneration.

Conclusions: These findings suggest that iCasp9-mediated elimination of iPSCs can effectively mitigate tumor risks while preserving their therapeutic potential for cartilage repair.

## Graphical abstract

**Graphical Abstract:**
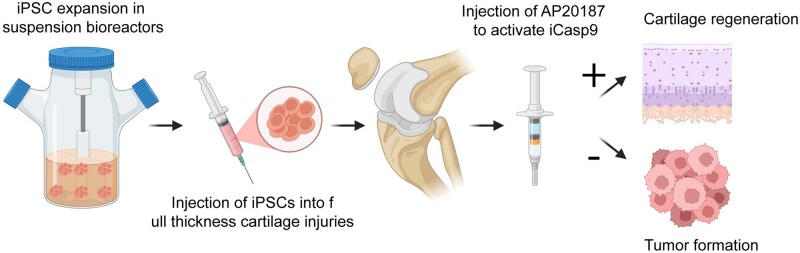

Significance Statement This study addresses a critical safety concern in induced pluripotent stem cell (iPSC) therapy for cartilage repair by demonstrating that the iCaspase9 suicide gene system effectively eliminates tumorigenic iPSCs while preserving regenerative potential. Current cartilage treatments are limited, and while iPSCs offer promise, tumor formation after transplantation has hindered clinical translation. By combining iCasp9 with *p21*-deficient iPSCs, this research provides a practical solution to prevent tumorigenesis without compromising therapeutic efficacy. The findings show complete tumor prevention in both wildtype and *p21*-knockout iPSCs while maintaining equivalent cartilage regeneration. This work represents a significant advance toward safe clinical application of iPSC-based therapies for cartilage injury and osteoarthritis.

## Introduction

Osteoarthritis (OA) is characterized by the gradual and ongoing deterioration of an entire joint, including the cartilage, muscles, and surrounding tissues. More than 500 million individuals were affected with OA globally in 2019, and this incidence of this insidious disease is only increasing. While affecting both sexes, this condition is more commonly observed in women than in men. It leads to reduced range of motion, edema, decreased flexibility, and persistent pain.^1^ OA is the second most widespread chronic disease globally, after hypertension. It is also the second leading cause of musculoskeletal disorders and disabilities.^2^^,^^3^

Articular cartilage is a connective tissue that overlays the bone surface within synovial joints. Due to its lack of blood vessels, lymphatic vessels, and nerves, articular cartilage demonstrates poor regenerative ability post-injury.^4^ Consequently, in certain instances, the healing process is biased towards fibrotic-like repair in place of regeneration in where the cartilage defect may initially fill through the production of fibrocartilage, which is of lower quality compared to articular cartilage tissue and demonstrates increased wear and tissue degradation breaks. Therefore, the current paradigm is that even small injuries to the articular cartilage surface can progress to full thickness cartilage loss and even progress to advanced stages of OA.^5^^,^^6^

The current treatment options for articular cartilage rely on the nature of the osteochondral lesion and can involve either conservative therapy or surgical procedures. Noninvasive methods such as continuous passive motion (CPM), electrical stimulation (ES), laser therapy, and medication may improve the process of cartilage restoration.^7^ Similarly, surgical techniques including microfracture, subchondral drilling, osteochondral grafts, and cell-based therapies can also be used in the attempt to improve joint function.^5^ Cell-based therapies, particularly autologous chondrocyte implantation (ACI), have been explored for localized cartilage repair and are considered among the few approaches showing potential for disease modification in focal cartilage lesions. While early clinical trials yielded mixed results, newer techniques and long-term follow-up studies have demonstrated improved outcomes.^8^ That being said, there are still no known therapies that can effectively and reproducibly slow, stop, or reserve the OA disease process once it has begun. In terms of cell therapies, mesenchymal stem cells have garnered significant attention, but due to many conflicting clinical studies, the overall evidence showing that these cells have any meaningful benefit for the treatment of cartilage injuries and/or OA is considered poor. It is important to note that OA can be broadly categorized into primary (idiopathic) and secondary forms. Primary OA is systemic and associated with aging and genetic predispositions, whereas secondary OA results from joint injuries, mechanical stress, or underlying conditions such as trauma or infection. The murine full-thickness cartilage defect (FTCD) model used in this study mimics aspects of secondary OA and post-traumatic cartilage injury, making it relevant for evaluating localized regenerative therapies rather than systemic disease modification.

The ability of pluripotent stem cells (PSCs) to proliferate indefinitely and differentiate into various cell types has captured the interest of the field of regenerative medicine. Implanting PSCs or PSC-derived chondrocytes into damaged articular cartilage is an ideal approach for treating OA and other skeletal disorders.^9^ However, the potential for tumor formation by direct transplantation of PSCs poses significant risks to PSC-based therapy.^10^ Therefore, it is imperative to eliminate the risk of tumor/teratoma formation prior to any clinical implementation.

Previous research has demonstrated that the introduction of murine embryonic stem cells (ESCs) or induced pluripotent stem cells (iPSCs) into osteochondral injuries in knee joints can result in the development of teratomas, which subsequently caused destruction of the knee structure and function.^11^ In another study, mouse ESCs were implanted into cartilage lesions in two groups of rats: one group with unrestricted joint movement and another group with immobilized joints^12^ with only the restricted group developing teratomas. When murine iPSCs with 100% genetic similarity to the donor were employed, this led to improved bone healing and cartilage regeneration; however, when mice received iPSCs with ∼70% genetic similarity, this resulted in the establishment of tumors instead of the intended bone and cartilage regeneration after 8 weeks post-transplantation.^13^ In another study that used a novel approach to target iPSCs to the defect using a magnetic label with an external magnetic field did not give rise to teratomas, whereas subjects not exposed to the magnetic field exhibited the development of teratomas 7 weeks after the transplantation.^14^ Overall, the variability of tumor/teratoma development within these studies and others clearly demonstrate the need for reproducible and robust approaches to inhibit any type of tumor formation post-transplant.

The introduction of suicide gene safety switches into cells has revolutionized cell-based therapy by enabling the elimination of transplanted cells in vivo.^15^ The iCasp9 system is a suicide gene that triggers apoptosis in cells that exhibit a genetically engineered caspase-9 that is dimerized by an exogenous drug. Administration of a nontoxic small-molecule drug class called chemical inducers of dimerization (CID), specifically AP20187, leads to the formation of dimers of iCasp9, triggering the activation of the intrinsic apoptotic pathway and initiating a series of caspase activities that result in cell death.^16^^,^^17^ The integration of iCasp9 safety guard into iPSCs can effectively mitigate the safety issue related to the propensity of residual undifferentiated and semi-differentiated iPSCs to form tumors upon being transplanted. While these gene systems are beginning to be explored in clinical trials, it has been shown that they are not 100% effective in triggering apoptosis. Therefore, we have recently demonstrated that attenuation of p21^(CIP1/WAF1)^ either genetically or pharmacologically enhances the functionality of iCasp9.^18^ As a cyclin-dependent kinase inhibitor, p21 is a cell cycle regulator that exhibits a paradoxical behavior, acting either as a tumor suppressor gene or an oncogene depending on the cellular context.^19^ When DNA is damaged or cells experience stress, it acts as a tumor suppressor gene by inducing cell cycle arrest in the nucleus.^20^ However, p21, can also function as an oncogene by binding to caspase3 in cytoplasm and preventing apoptosis.^21^ Therefore, its absence releases this inhibition of caspase3 and thereby enhances apoptosis in the iCasp9 system.

Another interesting aspect of p21 is that it have been previously implicated in endogenous mammalian regeneration—including spontaneous cartilage regeneration. This was first observed in the MRL mouse, which has exceptional proficiency in the healing and regeneration of many tissue systems, including the corneal epithelium, digit tips, and partial healing of lower phalanges.^22^ Additionally, it exhibits the ability to regenerate articular cartilage, a process that other mammals are unable to regenerate. MRL mouse cells exhibit reduced p21 level and cell cycle arrest in the G2/M phase.^23^ Similarly, adult *p21*^−/−^ mice, like MRL mice, also have cell cycle arrest in the G2/M phase, accompanied with regenerative responses at the morphological and histological levels, with minimal scarring.^23^^,^^24^

Taken together, this led us to the question if undifferentiated iPSCs were injected into the injured joint, could these cells regulate cartilage regeneration and would the iCasp9 strategy reduce the risk of tumor formation post-transplant? Since cartilage is largely comprised of extracellular matrix, it is an ideal target for iCasp9 abrogation strategies, because exogenous cells can be killed afterwards with minimal effects on regenerated tissues. We demonstrated that the in vivo iCasp9-induced cell death had a 100% success rate, as none of the animals treated with AP20187 developed teratomas/tumors. There were no disparities observed in the repair of cartilage injuries between mice who received iCasp9 engineered *p21*^−/−^ or *p21*^+/+^ iPSCs showing that both genetically engineered iPSC lines demonstrated both safety and efficacy.

## Results

### In vitro chondrogenesis

Prior to transplantation, we evaluated the ability of *p21*^−/−^ and *p21*^+/+^ iCasp9 transgenic (T)iPSCs to undergo into chondrogenesis via in vitro directed differentiation. After developing embryoid bods (EBs), the cells were subjected to treatment with chondrogenesis medium, and after 17 days of exposure, the cells were harvested and assayed for qRT-PCR. Expression of chondrocyte markers *Col2a1* (A), *Sox9* (B), and pluripotency marker *Oct4* (C) were analyzed throughout the differentiation process. Both *p21*^−/−^ and *p21*^+/+^ TiPSCs exhibited the ability to undergo chondrogenesis based upon *Col2a1* and *Sox9* expression (Figure S1). Except for the undifferentiated TiPSC samples, both genotypes exhibited a deficiency in *Oct4* expression during in vitro differentiation.

### In vivo tumor ablation

**Figure 1.**
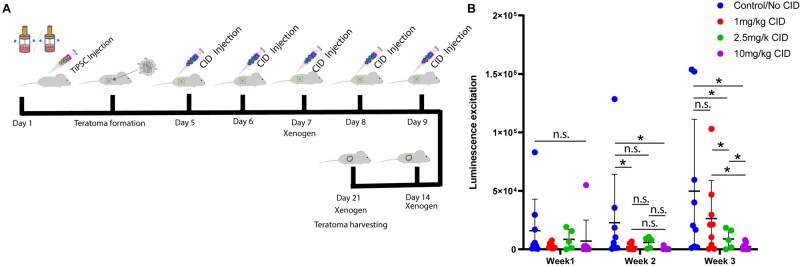
Teratoma ablation. Schematic diagram showing experimental steps (A). In vivo apoptosis assay with SCID mice. Quantified data of luminescence intensity emitted from tumor weekly measured in carrier control/no AP20187 *n* = 9, 1 mg/kg *n* = 9, 2.5 mg/kg *n* = 9, 10 mg/kg *n* = 9. *P < .05, ns = non-significantly different.

Prior to in vivo cartilage repair experiments, we evaluated the efficacy of iCasp9 in ablating TiPSCs in vivo (Figure 1). Teratomas were generated and ablated with varying concentrations of AP20187 to determine the optimal concentration of AP20187 to prevent teratoma formation by promoting apoptosis. Subcutaneous injections of *p21*^−/−^ or *p21*^+/+^ TiPSCs were used to induce teratomas in mice. Five days post-TiPSC transplantation AP20187 was injected for five consecutive days at one of three doses. The doses adhered to the minimum (1 mg/kg) and maximum recommended (10 mg/kg) dosages for the drug with an additional dose at 5 mg/kg. Teratoma size was evaluated over a course of 3 weeks using repeated measures noninvasive Xenogen-based bioluminescence imaging. The measured luminescent emission from SCID mice (*n* = 9 in the carrier control group, *n* = 9 in the 1 mg/kg AP20187 injection group, *n* = 9 in the 2.5 mg/kg AP20187 injection group, and *n* = 9 in the 10 mg/kg AP20187 injection group) revealed that the size of teratomas increased in the control group, while some cells were able to escape AP20187 treatment and induce teratomas but weeks post-injection in the 1 and 2.5 mg/kg groups (Figure 1). In the group that received 10 mg/kg AP20187, only background levels of luminescence was detected (Figure 1). These results demonstrated that the iCasp9 system successfully inhibited tumor formation in an AP20187 dose–response relationship in vivo.

### Cartilage repair

To assess the ability of the TiPSCs to repair injured cartilage and determine if treatment with the AP20187 could inhibit tumor/teratoma formation in vivo within the joint environment, we performed a standardized full-thickness cartilage defect (FTCD) in mice commonly used by our lab^24-27^ followed by transplantation of TiPSCs.

A control group consisting of *n* = 8 mice did not receive any injury (sham), AP20187 injections, nor TiPSC transplantation. In healthy articular cartilage, chondrocytes exhibit an elliptical shape on the surface and transition into a rounded shape as they move deeper (Figure S2). A further *n* = 8 mice underwent FTCD without any TiPSCs nor AP20187 injected into the joint (Figure S2). With this type of injury, there is always a range of healing outcomes and we have presented the best and poorest outcomes within the group of mice, but as expected there was minimal endogenous cartilage repair observed (Figure S2).

**Figure 2.**
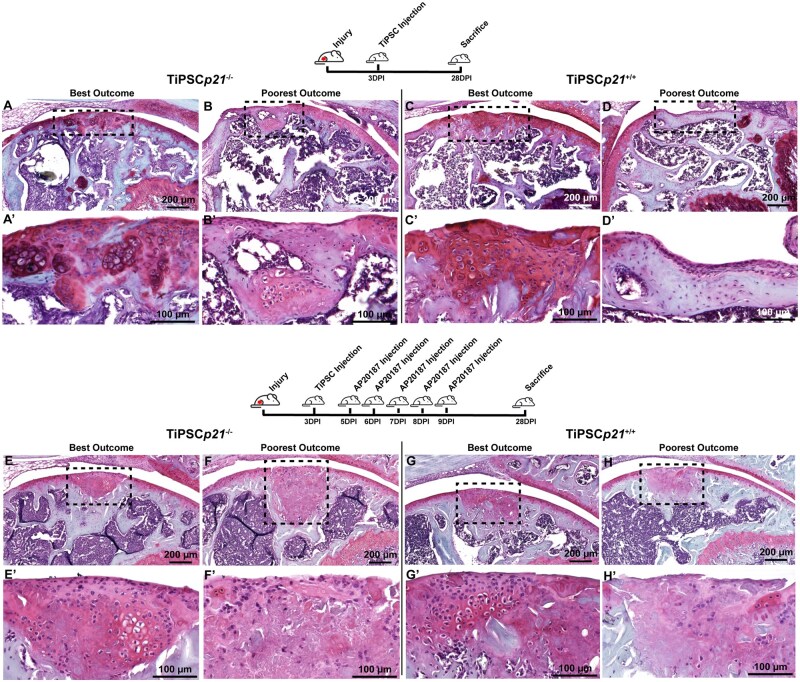
TiPSC transplantation into FTCD of mice knee joints with or without AP20187. Safranin-O-stained histology sections of injured mice knee joints injected with TiPSCs*p21*^−/−^ (A, B, E, F) or TiPSCs*p21*^+/+^ (C, D, G, H) with (E–H) or without (A–D) AP20187. The dashed black box highlights the injury site within the knee joint and images denoted with “ʹ” show a higher magnification of that site.

**Figure 3.**
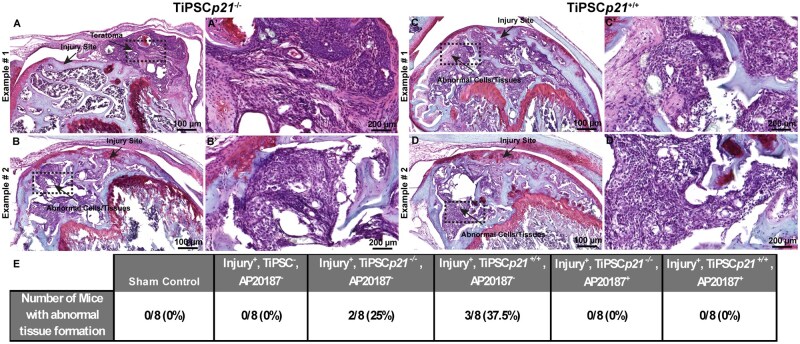
TiPSC transplantation into FTCD of mice knee joints can lead to abnormal tissue formation. Safranin-O-stained histology sections of injured mice knee joints injected with TiPSCs*p21*^−/−^ (A, B) TiPSCs*p21*^+/+^ (C, D) in the absence of AP20187 can lead to the formation of abnormal tissues and/or tumors in the joint environment. The number of animals with any indication of abnormal tissue formation was quantified (D).

To evaluate the regenerative capacity of TiPSCs in this injury model, mice knees were implanted with *p21*^−/−^ or *p21*^+/+^ TiPSCs 3 days post-FTCD, and no AP20187 was delivered to this group of mice (Figure 2). The morphology of the regeneration area cells is predominantly comprised of chondrocytes in the examples of best healing outcomes with either TiPSC line. However, while chondrocytes were observed within every injury site of animals treated with *p21*^−/−^ TiPSCs, in some animals treated with *p21*^+/+^ TiPSCs, fibrocartilage and/or bare bone was observed within the injury site (Figure 2). Another important point to note was that we did observe the presence of abnormal tissue/cell and teratomas generated by both *p21*^−/−^ (37.5% of mice) and *p21*^+/+^ (25% of mice) TiPSCs in animals that did not receive AP20187 (Figure 3). Yet, it is also important to note that in the rare occasion that tumor/teratoma formation was present, we still observed some level of cartilage regeneration within the defect site (Figure 3).

### Quantification of cartilage repair

This study utilized the 14-point histological scoring system developed by Fitzgerald et al.^28^ to quantify the level of cartilage repair post-FTCD. This system evaluates cell morphology, matrix staining, surface regularity, thickness of cartilage, and integration with native cartilage. However, to account for any abnormal tissue formation due to the injection of TiPSCs, we have added a factor: ectopic cartilage development rated on a scale of 0 to 3. Therefore, completely normal cartilage would have a score of 17 and the absence of cartilage would result in a score of 0. The full scoring system is presented in Figure S3.

**Figure 4.**
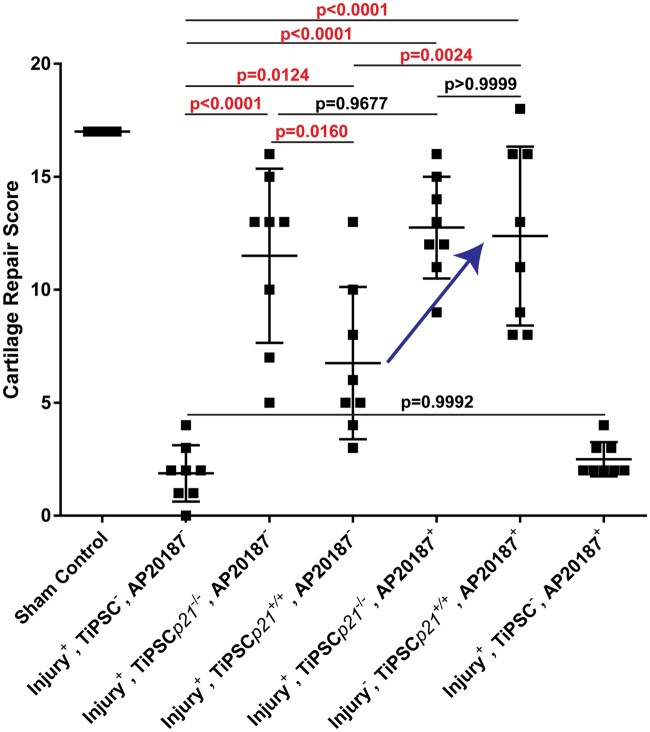
Quantification of cartilage repair. Safranin-O-stained histology sections from mice in all treatment groups were graded and quantified. Increased cartilage repair was observed in all mice that received TiPSCs*p21*^−/−^ or TiPSCs*p21*^+/+^ with or without AP20187. The blue arrow highlights the significant increase in cartilage repair observed when TiPSCs*p21*^+/+^ transplanted mice also received AP20187. The *P* values are presented in the figure and values lower than .05 are considered significant (red text).

When all the samples were histologically graded and compared, it was found that the injured, untreated mice did not demonstrate any meaningful level of endogenous cartilage repair (Figure 4). When TiPSCs were introduced into the injured joint, a significant increase in cartilage repair was observed with both *p21*^−/−^ and *p21*^+/+^ TiPSCs in the absence of AP20187. Furthermore, a significant increase in cartilage repair was observed in mice that received *p21*^−/−^ vs. *p21*^+/+^ TiPSCs (Figure 4). Interestingly, when the mice were treated with AP20187, a significant increase in cartilage repair was observed in mice receiving *p21*^+/+^ TiPSCs vs. mice that received the same cell line but without AP20187 (Figure 4, blue arrow). This led us to examine an additional control group to determine whether AP20187 had any impact on cartilage repair and we were able to determine that this is not the case. Therefore, immunofluorescence imaging was undertaken to determine whether the transplanted iPSCs were present in the injury site and whether there were any differences in localization/behavior in the presence of AP20187.

### Immunofluorescent detection of transplanted cells

**Figure 5.**
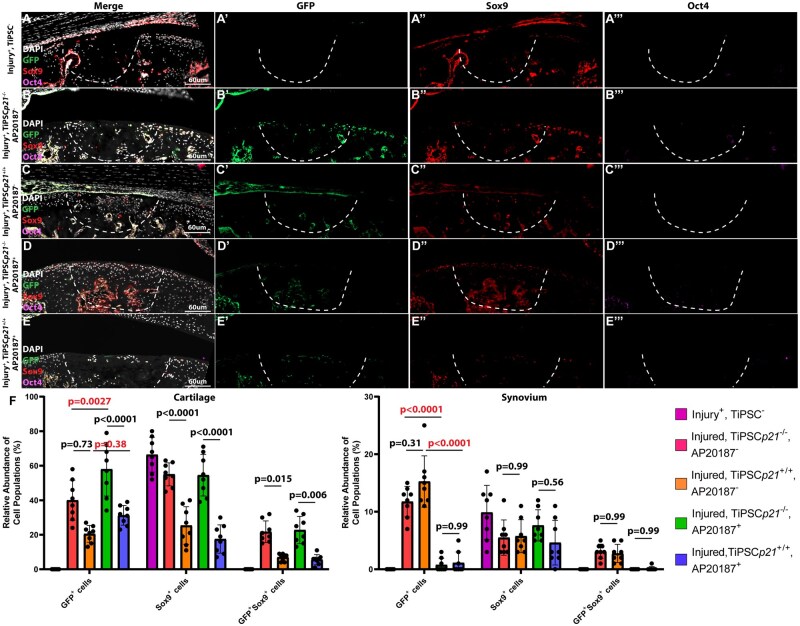
Quantification of iPSCs in the FTCD site. Histology sections from mice in all treatment groups (A–E) were stained for GFP (green, ‘), Sox9 (red, ‘’) and Oct4 (violet, ‘’’). Tissue cytometry was used to quantify the GFP, Sox9, and GFP-Sox9 double-positive cells (F). The *P* values are presented in the figure and values lower than .05 are considered significant. The *P* values in red text highlight the comparisons between GFP^+^ cells with or without AP20187 treatment in the cartilage (left) or synovium (right).

Histological sections of the injury site within all mice groups were stained for GFP (TiPSCs), Sox9 (chondrocyte marker), and Oct4 (core pluripotency factor). When only PBS (no cells) was injected into the injured joint, no GFP or Oct4 staining was detected. Sox9 staining was detected adjacent to the FTCD site with minimal staining present within the defect site itself (Figure 5A). When TiPSCs*p21*^−/−^ were injected without AP20187, GFP and Sox9 staining was observed within the FTCD site while no Oct4 staining was observed (Figure 5B). When the TiPSCs*p21*^+/+^ were injected in the absence of AP20187, minimal GFP, Sox9, and no Oct4 staining was observed within the FTCD site (Figure 5C). In the presence of AP20187, both TiPSCs*p21*^−/−^ and TiPSCs*p21*^+/+^ were observed within the defect site with no presence of Oct4 expression (Figure 5D and E). Tissue cytometry was employed to quantify the GFP^+^, Sox9^+^, and GFP^+^Sox9^+^ expressing cells in the defect site (Figure 5F). Overall, there were more TiPSCs*p21*^−/−^ cells present in the FTCD defect site with or without Ap20187 vs. TiPSCs*p21*^+/+^. Furthermore, TiPSCs*p21*^−/−^ also demonstrated increased in vivo differentiation into chondrocytes vs. TiPSCs*p21*^+/+^ as evidenced by the increased GFP^+^Sox9^+^ expressing cells in the FTCD site (Figure 5F). There was actually an increase of TiPSCs*p21*^−/−^ in the FTCD site with AP20187 treatment and no difference in the number of TiPSCs*p21*^+/+^ in the defect site with AP20187 (Figure 5F). This led us to question if the iCasp9 was working properly and therefore also decided to quantify the GFP+ cells within the synovium since we were able to identify iPSCs in this tissue with the immunofluorescence (Figure 5B and C). The transplanted iPSCs (either TiPSCs*p21*^−/−^ or TiPSCs*p21*^+/+^) were almost completely lost with the treatment of AP20187 suggesting that the iCasp9 and AP20187 were functioning properly.

## Discussion

PSCs can divide indefinitely and differentiate into any cell/tissue type in the body, making them an excellent source of treatment for a wide range of diseases and injuries; particularly if we consider diseases or injuries in which multiple cell types may be required to effect repair. Nevertheless, there are still unresolved challenges that need to be addressed before proceeding the clinic. Malignant transformation of PSCs can develop because of the presence of undifferentiated, partially differentiated, or even undesired cell types. While PSCs are undergoing pre-clinical trials to investigate articular cartilage repair, these studies typically employ the differentiated progeny of the PSCs such as MSCs or chondrocytes. An argument can be made that we don’t exactly know what cell type(s) are required for cartilage regeneration and therefore by using a pluripotent cell that can take on any phenotype as necessary in the micro-environment may have advantages. This approach would also come with increased risks as the tumorigenicity of PSC transplants in knee injuries has not been adequately addressed.

The implementation of a cell suicide technique has been shown to effectively eradicate specific cells both in vitro and in vivo,^15-17^ thereby reducing the associated risks and broadening the clinical use of PSCs. The iCasp9 system, used in this study, has the ability to specifically remove transplanted cells following the administration of AP20187.^29^^,^^30^ Previous studies have utilized this iCaspase9 technology to eliminate teratomas originating from mice in live organisms.^15^^,^^31^ In the current study, we were able to effectively eliminate teratomas in immune-compromised mice at a 10 mg/kg dose (but not at 1 mg/kg or 2.5 mg/kg). While the lower doses did show a reduction/trend in decrease of teratoma size, they were not nearly as effective as the maximum dose and were not selected to move forward for the cartilage repair experiments.

In this study, we not only added the iCasp9 transgene into the cells, but additionally investigated if the absence of p21 could augment repair. We and others have shown the p21 knockout mice demonstrate increased tissue regeneration including articular cartilage repair^23^^,^^24^^,^^32-34^ and that there is an inverse relationship between chondrogenesis and p21 expression.^35-37^ Before employing the wild-type and knockout *p21* iPSCs in vivo, we first examined their in vitro chondrogenic potential and found that while both iPSC lines effectively differentiated into chondrocytes, there was no increased chondrogenesis observed in the *p21*^−/−^. This would appear to be contradictory to the published literature, but it is important to recognize that we only assayed differentiation through *Col2a* and *Sox2* mRNA expression and didn’t quantify pellet size or GAG content. Therefore, it is possible that iPSCs lacking may show differences in chondrogenic potential if additional outcome measures were employed; however, in this experiment, we simply wanted to demonstrate that the transgenic modification of these iPSCs didn’t reduce their inherent chondrogenic ability.

Our in vivo chondrogenesis experiments were informed by previous observations suggesting undifferentiated PSCs can repair cartilage defect, provided the cells remain within the joint capsule and the cell numbers are not too high.^38^ One study also demonstrated that teratoma formation in FTCDs could be prevented by allowing mice to move freely following transplantation instead of immobilization.^12^ Here, it was suggested that cyclic loading might promote PSC cartilage differentiation, thereby suppressing teratoma formation. Leveraging from this published literature, we designed an experiment to investigate the ability of *p21*^−/−^ and *p21*^+/+^ TiPSCs to effect cartilage regeneration in a FTCD murine model. Contrary to the previously mentioned study, we did observe tumors/abnormal cell growth in ∼40% of mice injected with *p21*^−/−^ TiPSCs and 25% of mice injected in the *p21*^+/+^ TiPSCs in the absence of AP20187. One reason might be that we employed iPSCs while the previous study used ESCs.^12^ Also, we only actually observed one clear tumor/teratoma in all the mice examined and the other abnormalities where morphologically undifferentiated cells within the subchondral bone marrow, which was not commented in the previous study and may have been missed and/or since the other study used mouse ESCs transplanted into rats, this could have resulted in additional confounding variables. Yet, even though we detected these abnormalities in some mice, all mice within the AP20187^-^ groups demonstrated some level of cartilage regeneration. We suggest this is a critical point demonstrating efficacy even in cases where safety was not obtained. Interestingly, we did observe an increase in cartilage regeneration in *p21*^−/−^ TiPSCs vs. *p21*^+/+^TiPSCs but only in the absence of AP20187. Given that the main difference we observed in the joint space between mice treated with or without AP20187 was the number of iPSCs that had taken up residence in the synovium, this could infer that the *p21*^−/−^ TiPSCs could be important in the additional benefits to cartilage repair observed in these mice. This concept is supported by the literature which has shown that tissue resident progenitor cells present in the synovium can effect/contribute to cartilage repair.^39-42^

Transplanted iPSCs were observed within the FTCD site as well as the adjacent synovium, and it is likely that they also integrated into other joint tissues. It was interesting to see that transplanted iPSCs were retained within the defect site post-AP20187 treatment, although this isn’t completely unexpected as the cartilage is completely avascular and receives much of its nutrients from the synovium/synovial fluid. Since the synovial fluid is a derivative of the plasma component of blood and the small volumes of synovial fluid in a mouse joint (>2 µL), it would be difficult to determine the AP20187 concentration in the fluid and to what extent we would have to increase the systemic dose to achieve the optimal concentration within the joint. Alternative, injecting AP20187 directly into the joint could be a way to increase levels and iCasp9 activation within cells in the cartilage.

This study investigated the use of iPSCs with the iCasp9 suicide gene and p21 knockout modifications for cartilage regeneration in a murine full-thickness cartilage defect (FTCD) model. While iPSCs demonstrated chondrogenic potential and the iCasp9 system effectively eliminated tumorigenic cells, p21 knockout did not significantly enhance in vitro chondrogenesis but showed increased chondrogenic differentiation in vivo. Tumor/abnormal cell formation occurred in some cases but was completely mitigated by iCasp9 activation. Further studies should focus on optimizing iPSC doses and investigating the effects of directly injecting AP20187 into joints to improve tumor suppression while maximizing cartilage regeneration efficiency and safety.

## Materials and methods

### Ethics statement

Animal experiments were carried out in accordance with the guidelines set by the Canadian Council on Animal Care. The animal protocol and surgical procedures AC20-0042 and AC19-0134 in this study received approval from the University of Calgary Health Sciences Animal Care Committee.

### Cell culture

iCasp9 transgenic, *p21*^−/−^ and wildtype murine iPSCs have been reported previously.^18^ iPSCs were cultured on MEFs using MEFs media consisting of high-glucose DMEM (Gibco, Cat: 11965118), 10% FBS (Gibco), 1× MEM Non-Essential Amino Acids (Gibco), and 50 units/mL Penicillin-Streptomycin (Gibco). After a period of 2 days, iPSCs were introduced into stirred suspension bioreactors (Stemgent Inc./Reprocell ABBWVS03A-6) at a concentration of 5 × 10^4^ cells/mL for iPSCp21^−/−^ and 1 × 10^5^ cells/mL for iPSC*p21*^+/+^. The bioreactors were set to rotate at a speed of 100 rpm, while the stem cell media consisted of high-glucose DMEM (Gibco), 15% FBS (Gibco), 1 mM Sodium Pyruvate (Gibco), 1× MEM Non-Essential Amino Acids (Gibco), 50 units/mL Penicillin-Streptomycin (Gibco), 0.1 mM 2-Mercaptoethanol (Gibco), and 100 U/mL LIF (ESGRO^®^ Millipore-Sigma). Cell dissociation was performed enzymatically using TrypLE™ (Gibco).

### In vitro chondrogenesis

iCasp9 transgenic, wildtype, and *p21*^−/−^ iPSCs were cultured in bioreactors for a duration of 4 days. Next, the suspended droplets of embryoid bodies (EBs) were created by using a pipette to dispense 4 × 10^4^ iPSCs in 20 μL of embryonic stem cell (ESC) media without leukemia inhibitory factor (LIF) onto the inner surface of a 100 mm dish lid. Following a 4-day incubation at a temperature of 37 °C and a carbon dioxide concentration of 5%, each embryoid body (EB) was moved into a 0.2 mL VWR^®^ PCR 8-Well Tube Strip (VWR) containing 50 μL of chondrogenic media. The chondrogenic medium was replaced every other day for a period of 30 days. The chondrogenic media consisted of DMEM, 1× Antibiotic-Antimycotic (ThermoFisher), 1× GlutaMax^TM^ (ThermoFisher), and 1× MEM-NEA (Gibco). The media also contained ascorbic acid (Sigma), BMP-2 (Peprotech, Catalogue Number 120-02), TGF-β3 (Peprotech, Catalogue Number 100-21), Dexamethasone (Sigma, Catalogue Number D2915), sodium pyruvate (Gibco Cat. No.11360-070), and ITS (BioWhittaker). NaOH was used to adjust to pH to 7.

### Real-time PCR

The chondro-aggregates, collected at 4-day intervals, were lysed using 100 μL of TRIzol™ Reagent (ThermoFisher). Lysed cells were utilized for the isolation of RNA and the synthesis of cDNA. The RNA extraction was carried out using the RNA extraction kit (E.Z.N.A.^®^ Total RNA Kit I, Omega Biotek). The RNA extraction process was conducted following the instructions provided by the manufacturer. The cDNA was synthesized from the extracted RNA using the High-Capacity cDNA Reverse Transcription Kit (ThermoFisher) following the manufacturer’s instructions. RT-PCR was conducted using TaqMan probes (ThermoFisher) to detect the presence or absence of specific markers. The markers tested were Oct4 and Sox9 to confirm the absence of pluripotency and Col2a to verify chondrogenesis. 18 s was used as a housekeeping gene.

### In vivo iCasp9 system functionality

The efficacy of the iCasp9 system was assessed using the Teratoma Assay described previously. A total of 10^6^ cells per 100 mL of DPBS were injected subcutaneously into the lower left back of immunocompromised mice. Starting on day 5, mice were injected intraperitoneally with AP20187 at a specific dose for five consecutive days. The AP20187 is mixed with pure ethanol to create a stock solution with a concentration of 62.5 mg/mL. The stock solution is prepared by mixing it with PEG-400 and Tween (2%) in water, following the manufacturer’s instructions and considering the AP20187 concentration for injection.

Before weekly Xenogen imaging, the mice were sedated using inhaled isoflurane at a concentration of 2.5%. They were also administered an injection of luciferin at a dose of 150 mg/kg in DPBS. After a duration of 15 min, the mice were examined for the presence of tumors using a Xenogen Imager.

### In vivo chondrogenesis and iCaspase9 system

Full-Thickness Cartilage Defects (FTCDs) were induced in mice by inserting a 27G needle into the trochlear groove, resulting in the formation of a localized cartilage defect measuring approximately 0.6 mm in diameter. A total of 2 μL of DPBS containing iCasp9 engineered *p21*^−/−^ or wildtype iPSCs at a concentration of 25 000 cells/μL were introduced into the knee joint space through interarticular injection 3 days post-FTCD. This procedure was carried out using a micro-manipulator connected to a 27-gauge Hamilton syringe, as previously described.^40^^,^^43^ For controls, sterile DPBS was used. Mice were intraperitoneally administered 10 mg/mL/kg mouse weight of CID for five consecutive days beginning on day 5 post-FTCD. Whole knee joints were harvested 4 weeks after FTCD and, following DPBS rinsing, fixed in Formalin Buffered 10% (Phosphate Buffer/Certified, Fisher Chemical™ Thermo Fisher Scientific, Cat. No. SF100-4). Subsequently, the joints were decalcified in EDTA 19%W/V pH 7.4 for a duration of 3 weeks. Throughout the initial week, the EDTA was altered daily. Every other day for the past 2 weeks, a new EDTA has been added. Tissue embedded in paraffin was sectioned to a 10 mm thickness, mounted on glass slides, and subjected to drying at 37 °C overnight after being placed on a heat plate at 45 °C–50 °C for 5 h. Proteoglycan was visualized using Safranin-O/rapid green staining in conjunction with a rapid green counterstain to distinguish bone.

### Immunofluorescence

Slides were subjected to immunostaining using antibodies that specifically bind to pluripotency markers Oct-3/4 (C-10), Alexa Fluor 647 (Santa Cruz), Sox2 (Novusbio), GFP (Abcam), and Hoeschst 33342 (ThermoFisher). A GFP antibody was used to track GFP expression because expression driven from the ROSA26 promoter was dim and was obfuscated by autofluorescence from surrounding bone. Immunostaining method followed the subsequent sequential steps. (1) Dewaxing and hydration, as previously explained. (2) The antigen retrieval technique entails utilizing 350 mL of a 10 mM sodium citrate solution with a pH of 6, heated to a temperature range of 60 °C–70 °C for a length of 1 h. Afterwards, the sample was rinsed twice with deionized water (dH_2_O) for a duration of 10 min each time. To prevent nonspecific binding, a 1:200 dilution of goat serum was made by adding 500 μL of goat serum to 100 mL of TPBS and incubating for 1 h. (5) The slides were exposed to the primary antibody and kept at a temperature of 4 °C overnight. (6) Following three washes with TPBS, the slides were subjected to overnight treatment with the secondary antibody. (7) Following three rounds of washing with TPBS and drying, coverslipping was performed using EverBrite™ Hardset Mounting Medium (Biotium, Cat.No.23003). The slides were scanned using the Axio Scan Z1 and the Zen3.5 (blue) software following a 2-h period.

### Tissue cytometry

Tissue cytometry (TissueQuest) was employed to quantify the immunofluorescent images. By utilizing automatic nuclear segmentation, Hoechst nuclear staining-stained cells were identified. To differentiate positive signals from autofluorescence induced by other markers and signals, thresholds and gates for the other markers were established using nonstained controls. Scattergrams and histograms were produced to visually represent the data associated with a range of metrics, such as signal intensity, total cell count, percentage of positive cells, and positive cells per area. In GraphPad Prism (Version 9.4.1), data were analyzed statistically via two-way ANOVA test for multiple comparisons. A *P*-value of .05 or less was deemed to indicate statistical significance. By calculating the mean of replicates, differences in outcomes among sample groups were assessed.

## Supplementary Material

szaf056_Supplementary_Data

## Data Availability

The data underlying this article will be shared on reasonable request to the corresponding author.
