# Supplementary material for: Teratoma-free cartilage regeneration using p21−/− iPSCs engineered with iCasp9
Source: Stem Cells Transl Med. 2025 Nov 19;14(11):szaf056. doi: 10.1093/stcltm/szaf056 (PMC12629534; doi:10.1093/stcltm/szaf056)
Supplement: szaf056_Supplementary_Data [file szaf056_supplementary_data.docx]

**Supplementary Information**

**Teratoma-free Cartilage Regeneration using *p21*^-/-^ iPSCs Engineered with iCasp9**

Leila Larijani^1^, Derrick Rancourt^1,2^, Roman J Krawetz^1,3*^

^1^McCaig Institute for Bone & Joint Health, University of Calgary, Calgary, Alberta, Canada.

^2^Department of Oncology, Cumming School of Medicine, University of Calgary, Calgary, Alberta, Canada.

^3^Department of Cell Biology and Anatomy, Cumming School of Medicine, University of Calgary, Calgary, Alberta, Canada.

Leila Larijani: llarijan@ucalgary.ca

Derrick Rancourt: rancourt@ucalgary.ca

* Corresponding Author:

Roman J Krawetz:

Faculty of Medicine, University of Calgary. 3330 Hospital Drive NW, Calgary, Alberta, Canada T2N 4N1. Tel: (403) 210-6268, Email: [rkrawetz@ucalgary.ca](mailto:rkrawetz@ucalgary.ca)

**Key words**: cartilage regeneration, iCasp9, induced pluripotent stem cells, tumor formation

**Running Head**: Cartilage Regeneration using iPSCs with iCasp9


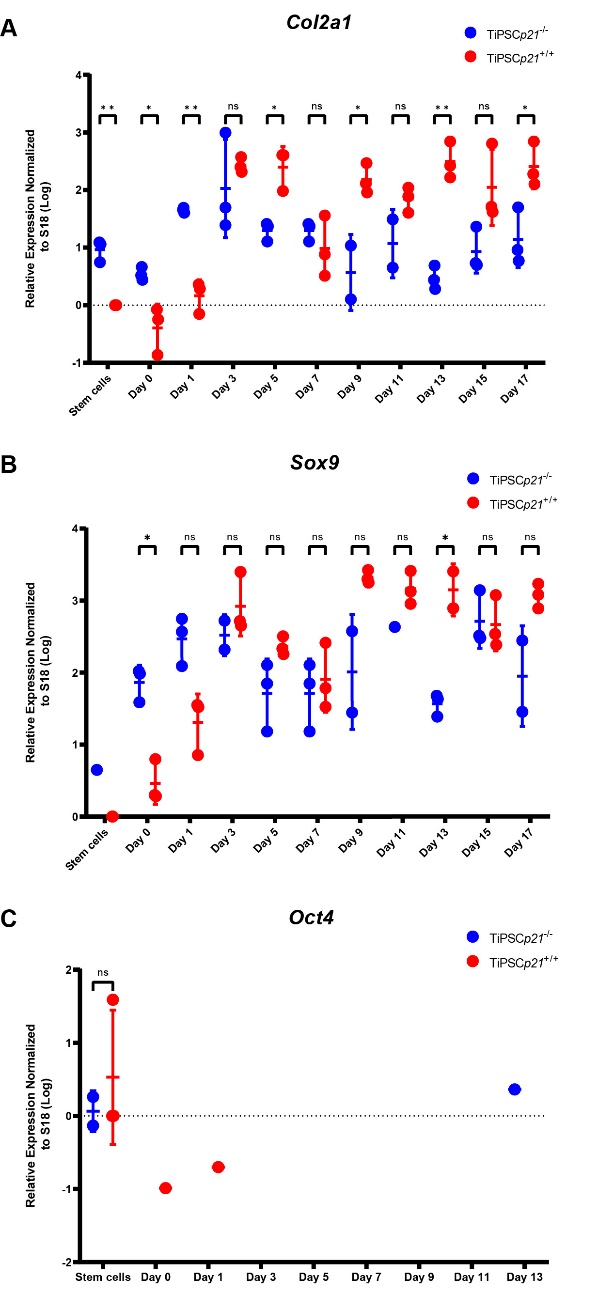


**Figure S1**: **Analysis of *in vitro* chondrogenesis.** Graphs show qRT-PCR results for chondrogenic marker gene expression profiles (A) *Col2a1*, (B) *Sox9*, in addition to pluripotency, *Oct4* (C). T-tests were performed in *p21*^-/-^ and *p21*^+/+^ iCasp9 for individual timepoints. * = p<0.05, ** = p<0.01, ns = not significantly different. All assays were performed using biological and methodological triplicates.


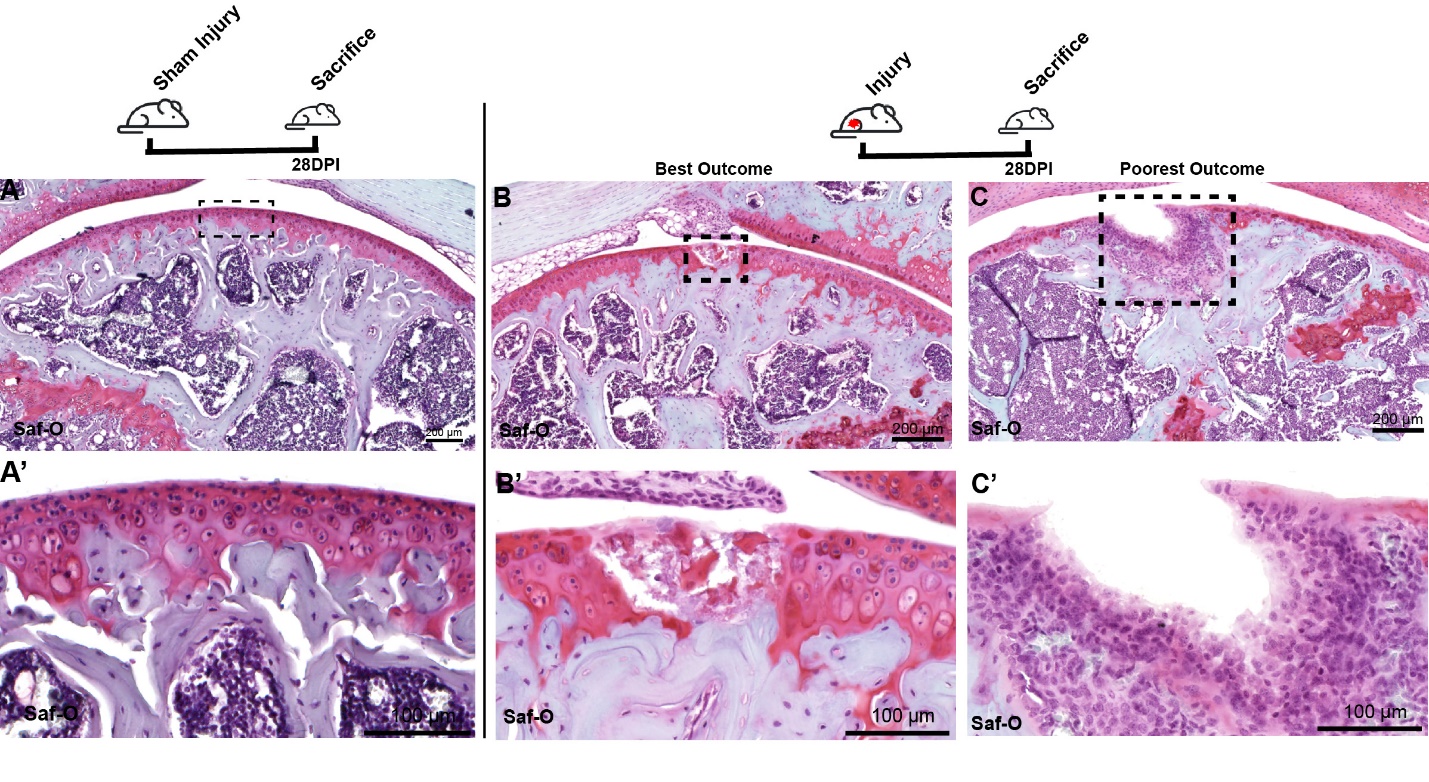


**Figure S2: Sham and FTCD healing outcomes.** Knee joints that underwent a sham injury (n=8) demonstrate no obvert changes to the articular cartilage (A, A’). Mice that underwent FTCD (n=8), without TiPSCs nor AP20187 show a range of healing outcomes at 28DPI (B, B’, C, C’). Black dashed box highlights the injury site.


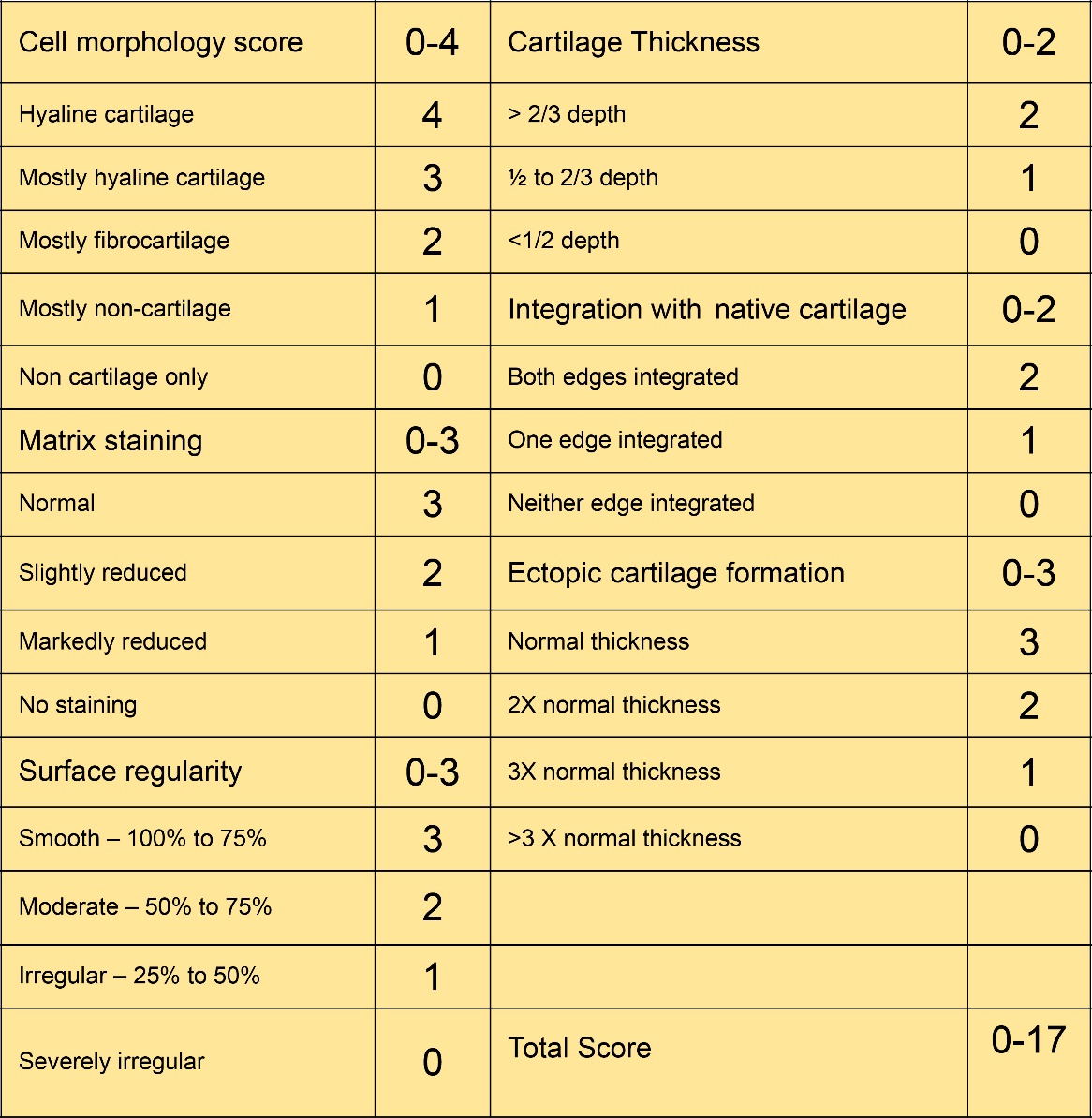


**Figure S3. Sample cartilage repair histological scoring sheet.** Completely normal cartilage would have a score of 0 and the absence of cartilage would have a score of 14 – if any ectopic cartilage formation was observed this could reduce the score to 17.
